# Prevalence and risk factors of sheep and goats fasciolosis in Ethiopia: A systematic review and meta-analysis

**DOI:** 10.1371/journal.pntd.0013074

**Published:** 2025-08-18

**Authors:** Simachew Getaneh Endalamew, Alebachew Tilahun Wassie, Andnet Yirga Assefa, Yihenew Getahun Ambaw, Solomon Mekuriaw Ayalew, Solomon Keflie Assefa

**Affiliations:** 1 Department of Veterinary Epidemiology and Public Health, School of Veterinary Medicine, Bahir Dar University, Bahir Dar, Ethiopia; 2 Department of Clinical Medicine, School of Veterinary Medicine, Bahir Dar University, Bahir Dar, Ethiopia; 3 Haramaya University, College of Veterinary Medicine, Dire Dawa, Ethiopia; 4 Department of Horticulture, College of Agriculture and Environmental Science, Arsi University, Assela, Ethiopia; 5 Department of Biostatistics and Epidemiology, Institute of Public Health, College of Medicine, and Health Science, University of Gondar, Gondar, Ethiopia; Jiangsu Institute of Parasitic Diseases, CHINA

## Abstract

**Background:**

Fasciolosis is a parasitic disease caused by liver flukes of the genus *Fasciola*, predominantly *Fasciola hepatica* and *Fasciola gigantica.* This zoonotic disease significantly impacts both livestock and human populations, particularly in areas with extensive agriculture and poor sanitation. Ethiopia, one of Africa’s leading sheep and goats producers, is highly affected by fasciolosis. However, despite its economic and public health importance, there is a lack of comprehensive and up-to-date evidence on the prevalence and risk factors of small ruminant (sheep and goats) fasciolosis. Therefore, the objective of this systematic review and meta-analysis (SRMA) is to estimate pooled prevalence and identify risk factors of fasciolosis among small ruminants in Ethiopia.

**Methods:**

This SRMA was conducted based on the Preferred Reporting Items for Systematic Reviews and Meta-Analyses (PRISMA) guidelines. A comprehensive systematic review was performed across five electronic databases (Google Scholar, Embase, PubMed, Web of Science, and ScienceDirect), with all database searches and registers inquiries finalized on November 26, 2024. A random-effect model was used to determine the pooled prevalence of fasciolosis in sheep and goats. Heterogeneity was assessed, and the source of variation was analyzed using subgroup, sensitivity analysis and meta-regression. Publication bias assessment and sensitivity analysis were also performed to ensure the robustness of the review. Funnel plots and Egger’s asymmetry tests were used to investigate publication bias.

**Results:**

A total of 33 studies containing 9,578 small ruminants were included in the meta-analysis. The overall pooled prevalence of fasciolosis was 32.25% (95% CI: 25.97–38.86%) with substantially high between-study heterogeneity (inconsistency index (I^2^)) = 97.3%, p < 0.01). Among the variables analyzed for heterogeneity, species, publication years, season of data collection, and regions of the study were significant predictors of heterogeneity The sub-group analysis showed that the prevalence of fasciolosis among sheep and goats was 37.18% (95% CI; 31.06–43.51%) and 12.76% (95% CI; 4.06–25.19%), respectively. According to the region-based subgroup analysis, studies taken from Amhara region had the highest prevalence of fasciolosis among small ruminants (43.99% (95% CI: 31.83–56.52%)).

**Conclusion:**

This study highlights fasciolosis as a significant threat to Ethiopian small ruminants. Policymakers and veterinarians should prioritize evidence-based control programs in regions with high disease burden, through seasonal deworming, pasture rotation to disrupt snail ecology, and improved veterinary access in underserved areas.

## Author summary

Fasciolosis is a neglected parasitic disease caused by liver flukes and affects both animals and humans. In Ethiopia, fasciolosis poses a significant burden to animal health and agricultural livelihoods. Despite this, there has been no recent national-level summary of the disease’s prevalence or factors contributing to its occurrence in small ruminants. In this study, we systematically reviewed 33 published studies and conducted a meta-analysis to estimate the overall burden of fasciolosis in small ruminants (sheep and goats). Our findings indicate that nearly one in three small ruminants (32.25%) is infected with fasciolosis, with infection rates being significantly higher in sheep (37.18%) than in goats (12.76%). The highest burden was observed in the Amhara region, with a prevalence of 43.99%.These findings underscore the urgent need for coordinated disease control efforts, including regular parasite monitoring, targeted treatment strategies, and improved management practices. Addressing fasciolosis requires collaborative action from veterinarians, livestock owners, policymakers, and researchers to mitigate its impact on food security, rural income, and animal welfare in Ethiopia.

## 1. Introduction

Fasciolosis, caused by the liver flukes *Fasciola hepatica* and *Fasciola gigantica*, is a globally significant parasitic disease affecting both livestock and humans [1,2]. The World Health Organization (WHO) classifies fasciolosis as a neglected tropical disease owing to its widespread impact in low- and middle-income countries, particularly those with poor sanitation and extensive agricultural activity [3]. Globally, more than 550 million ruminants, including cattle, sheep, and goats, are affected, leading to estimated annual economic losses exceeding $3.2 billion due to decreased productivity, liver condemnation, and reproductive failure [4–6].

In livestock, fasciolosis causes chronic illness that silently reduces productivity. Sheep and goats, which are crucial for food security and income in many rural communities, suffer from reduced weight gain, milk yield, and fertility [7]. The disease also has zoonotic potential, with humans becoming infected through the ingestion of water or vegetables contaminated with metacercariae at the infective larval stage [8]. This transmission occurs particularly in areas where humans live in close contact with infected livestock or share water sources contaminated with feces. Human fasciolosis, once overlooked, is increasingly being recognized as a contributor to hepatobiliary disease and malnutrition in endemic areas [9].

The life cycle of *Fasciola* species includes freshwater snails of the family Lymnaeidae as intermediate hosts [10]. These snails thrive in climate-sensitive habitats such as irrigation channels, marshy grazing lands, and riverbanks. Within the snail, the parasite undergoes asexual development before being released into the environment as metacercariae, which adhere to vegetation or contaminate the water. Transmission to definitive mammalian hosts, including livestock and humans, occurs when contaminated sources are ingested [11]. Climate factors, such as rainfall, humidity, and temperature, play a central role in shaping the transmission risk [12,13].

In Ethiopia, Africa’s leading producer of sheep and goats, fasciolosis is endemic and is a major veterinary and economic burden [14]. *F. hepatica* predominates in highland areas above 1800 meters, whereas *F. gigantica* is more common in lowland regions below 1200 meters, although overlap is possible in mid-altitude zones [15,16]. Traditional pastoral practices, seasonal flooding, limited veterinary services, and ecological conditions contribute to sustained transmission [13,15,17]. Goats, owing to their browsing habits, are believed to have lower acute exposure than sheep, but chronic infections still affect overall productivity and health [18,19].

Despite numerous localized studies over the past decade, there is a lack of consolidated national-level evidence on the burden and determinants of small ruminant fasciolosis in Ethiopia. Therefore, the objective of this study was to generate a comprehensive national estimate of fasciolosis prevalence among sheep and goats in Ethiopia and to identify epidemiological factors influencing its distribution.

This study provides essential insights for veterinary professionals, policymakers, and researchers, facilitating evidence-based improvements in diagnostic approaches, therapeutic strategies, and prioritization of targeted, region-specific interventions. These findings also align with global efforts to mitigate the impact of zoonotic diseases, underscoring the interconnectedness of animal health, sustainable agriculture, and public health resilience.

## 2. Methods and analysis

### 2.1. Development of the review method

This study employed the Condition, Context, and Population (CoCoPop) framework [20] to structure its design. The methodological approach was developed and reported in strict accordance with the PRISMA 2020 guidelines for systematic review protocols [21] (S1 Table). Prior to data collection, the study protocol was prospectively registered with PROSPERO (International Prospective Register of Systematic Reviews) on 21 February 2024 (Registration ID: CRD42024576654) with a focus on fasciolosis in sheep and goats. The analysis specifically evaluated the pooled prevalence of fasciolosis in these species as the primary outcome.

### 2.2. Inclusion and exclusion criteria

All cross-sectional studies published in peer-reviewed journals and gray literature that were conducted in Ethiopia were eligible for inclusion in this study. To ensure the incorporation of current and pertinent evidence on the prevalence of fasciolosis in small ruminants, only studies conducted after 2015 were included. Different study formats that reported the prevalence of fasciolosis in small ruminants, including peer-reviewed journal articles, Master’s theses, and dissertations published in English were considered.

Research articles were excluded for one of the following reasons: (a) reported the knowledge, attitudes, and practices of small ruminant fasciolosis (qualitative studies), (b) articles with insufficient information or records with missing outcomes of interest or poor-quality articles, (c) personal opinions, correspondence, letters to the editor, proceedings, and reviews. In addition, to enhance the study’s precision, articles with small sample sizes (less than 100) were excluded.

### 2.3. Searching strategy

Data was retrieved from five major academic databases, including Google Scholar (www.scholar.google.com), Embase (www.embase.com), PubMed (www.pubmed.ncbi.nlm.nih.gov), Web of Science (www.thomsonreuters.com), and ScienceDirect (www.sciencedirect.com). The final literature search was finalized on November 26, 2024. The MeSH terms and keywords combined with Boolean operators were used to retrieve all relevant articles from the registers and databases. The search included keywords related to the population (small ruminants, ovine, caprine, sheep, goat), condition (fasciolosis, *F. gigantica*, *F. hepatica*), and context (e.g., Ethiopia). Boolean operators such as “AND” and “OR” were also applied to combine terms effectively. The search strategy, initially developed for PubMed, was adapted for other databases and included the following framework: ((((((((((((Ethiopia[Text Word]) OR (Ethiopia[MeSH Terms])) AND (sheep[MeSH Terms])) OR (sheep[Text Word])) OR (goat[MeSH Terms])) OR (goat[Text Word])) OR (small ruminants[Text Word])) AND (fasciolosis[MeSH Terms])) OR (*F. gigantica*[Text Word])) OR (*F. hepatica*[Text Word])) AND (prevalence[Text Word])) OR (seroprevalence[Text Word])). Retrieved records were organized using EndNote reference management software, where duplicates were eliminated through manual verification.

### 2.4. Evaluation of the quality of studies and risk of bias assessment

The quality of the included studies was assessed using Joanna Briggs Institute’s critical appraisal tool, designed to evaluate prevalence studies [20]. The tool has nine items measured with four options: “yes,” “no,” “NA”, and “unclear”. The assessment of included studies was conducted by two independent teams: Team A (ATW, SGE, SMA) and Team B (SKA, YHA, AYA). These teams utilized a four-tier evaluation system (“yes,” “no,” “unclear” and “NA”) to appraise article quality. Disagreements between evaluators were resolved through collaborative discussions and consultations with a panel of expert researchers. In the scoring protocol, a value of 1 was assigned for each “yes” response, while “no” or “unclear” answers (including instances where information was not reported or deemed irrelevant) received 0 points. Cumulative scores for each article were calculated on a scale of 0–9. Based on these totals, studies were classified into three quality orders: high (7–9 points), moderate (4–6 points), or low (0–3 points) (S2 Table). For this systematic review and meta-analysis, only articles ranked as high or moderate quality were retained for final analysis.

### 2.5. Extraction of data from eligible papers

The initial search results passed relevance screening, with only studies meeting predefined inclusion criteria proceeding to the analysis phase. A standardized extraction template was utilized for data collection, implemented independently by two research teams: Team A (ATW, SGE, SMA) and Team B (SKA, YHA, AYA). Discrepancies in extracted information were resolved through consensus-based discussions between the groups. The template captured key study parameters including author name, publication year, geographic location, diagnostic method, sample size, climatic conditions, season of data collection, prevalence rates, and elevation data. All extracted information was systematically organized and cross-verified using Microsoft Excel (version 16.54) to ensure accuracy and consistency throughout the review process.

### 2.6. Data synthesis and Statistical analysis

Data extraction focused on compiling proportions, standard errors, and 95% confidence intervals (CIs) from studies reporting relevant findings. For studies lacking precomputed 95% CIs, these intervals were derived using validated statistical formulas [22]:

$LCI=\;e^{\text{ln}(p)-1.96\sqrt{\frac{1}{E}}}\text{ While, }UCI=\;e^{\text{ln}(p)+1.96\sqrt{\frac{1}{E}}}.$

Here, *p* represents the proportion of fasciolosis cases, *E* denotes the total count of identified *Fasciola*, and LCI/UCI define the interval bounds. This approach ensured methodological consistency and accuracy in estimating uncertainty across heterogeneous datasets.

Statistical analyses were conducted using R Studio (v4.4.2) [23] with the “Meta” package (v8.0-1) [24]. Model selection (fixed vs. random effects) was guided by heterogeneity assessment, where a random effects framework was adopted due to significant between-study variability (I² = 97.3%). The double arcsine transformation (PFT) demonstrated the most appropriate fit for the assumption of normality, as evidenced by the Shapiro-Wilk test (W = 0.95291, p-value = 0.1618). Therefore, it was chosen to normalize the data and stabilize the variance estimation for the final meta-analytic model.

A random-effects meta-analysis was conducted to account for variability across studies, with variance estimation performed through the DerSimonian and Laird approach [25]. PFT-transformed prevalence rates of small ruminant fasciolosis and associated standard errors were calculated for each study using the inverse variance method, facilitating pooled estimates [22,26]. To enhance uncertainty quantification, confidence intervals were adjusted via the Hartung-Knapp-Sidik-Jonkman (HKSJ) procedure [27,28]. The extent of between-study heterogeneity was further assessed by computing confidence intervals for τ² (tau-squared) and τ (tau) using the Jackson method, which quantified both the magnitude and precision of variability among included studies [29].

The distribution of fasciolosis prevalence among small ruminants was graphically synthesized using a forest plot, displaying individual study estimates with 95% confidence intervals (CI). Between-study heterogeneity, attributable to methodological diversity, was quantified using the Cochrane Q-test [30], I² statistic (proportion of total variability due to heterogeneity rather than sampling error) [31], and prediction intervals to estimate the potential range of true effects [32].

Furthermore, subgroup analysis, sensitivity analysis, and meta-regression analysis, were employed to address the substantial heterogeneity observed in the pooled estimate derived from the random-effects model. These methods help to explore potential sources of heterogeneity and provide insights into the robustness of the findings. Subgroup analysis was conducted based on sample size, geographic region, animal species studied, season, publication year, and altitude to explore sources of variability. Sensitivity analyses assessed the reliability of combined results by systematically removing each study one at a time. If the confidence interval of the excluded study did not include the overall effect size estimate, it was considered to have a significant impact on the results [33].

Baujat plots were additionally employed to identify studies that had a significant impact on overall variability. The plot shows the contribution of each study to the overall heterogeneity (as measured by Cochran’s Q) on the horizontal axis and its influence on the pooled effect size on the vertical axis [34]. To identify factors influencing variability, season, publication year, sample size, and region were analyzed using both univariable and multivariable meta-regression models within a random-effects structure. Finally, publication bias was assessed by checking the symmetry of funnel plots and performing Egger’s regression test [35]. A non-significant outcome in Egger’s test (*p* > 0.05) suggested no evidence of significant publication bias or small-study effects.

## 3. Results

### 3.1. Study selection and identification

**Fig 1:**
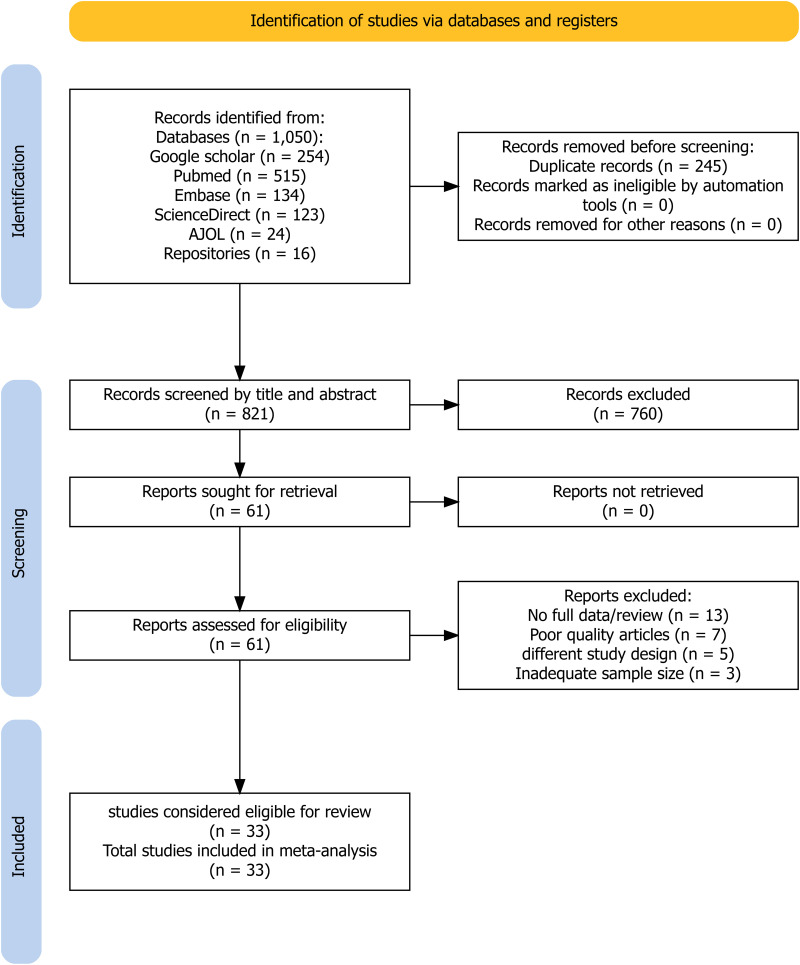
PRISMA flow diagram for study selection (identification, screening, eligibility assessment, and inclusion of studies) in the systematic review and meta-analysis.

A total of 1,066 studies were initially identified and screened, comprising 1,050 retrieved from electronic databases and additional 16 studies obtained from institutional repositories. Of these, 245 duplicate records were removed, and 760 studies were excluded after screening titles and abstracts due to irrelevance to the study objectives. Additionally, 28 studies were excluded for reasons such as not reporting the full outcome/reviews, poor quality, and inadequate sample size. Finally, 33 studies were considered suitable for inclusion in the qualitative and quantitative syntheses, as depicted in the PRISMA flow diagram (Fig 1).

### 3.2. Description of included studies

This SRMA incorporated data from 33 studies conducted between 2015 and 2024 within field, clinical and abattoir settings, comprising a total sample of 9,578 small ruminants. Among these, 3,419 individuals (32.25%) exhibited confirmed fasciolosis disease. Between 2015 and 2024, the Oromia and Amhara region accounted for the majority of studies documenting small ruminant fasciolosis in Ethiopia. Most of the studies were conducted on ovine species. The sample sizes varied from 121 to 576 small ruminants. The apparent prevalence of the disease ranged from 5.80% to 70.2% in the study areas (Table 1).

**Table 1:** Characteristics of the included studies and prevalence of fasciolosis among small ruminants in Ethiopia, 2024 (n = 33).

Authors with publication years	Species	No. of Examined	No. of Positive	Prevalence with 95% CI	Diagnosis method used
Abaya, S. W., et al. (2023a) [36]	Ovine	195	85	43.58 (35.24; 53.91)	Immunological test
Abaya, S. W., et al. (2023b) [36]	Ovine	141	13	9.21 (5.35; 15.87)	Immunological test
Abdurahaman, M., et al. (2019) [37]	Ovine	285	150	52.63 (44.84; 61.76)	Coproscopy
Alemu, B. and D. Chala (2019) [38]	Ovine	384	151	39.32 (33.53; 46.13)	Coproscopy
Amsalu, T. (2017) [39]	Ovine	576	61	10.59 (8.23; 13.62)	Coproscopy and Postmortem
Asrede, T. and A. Shifaw (2015) [40]	Ovine	390	198	50.76 (44.16; 58.36)	Coproscopy
Ayele, Y. et al. (2018) [41]	Ovine	280	170	60.71 (52.24; 70.57)	Coproscopy and Postmortem
Bayu, A. and S. Derso (2015) [42]	Ovine	384	170	44.27 (38.09; 51.45)	Coproscopy
Bedada, H. and F. G. W. Negash (2017a) [43]	Ovine	129	33	25.58 (18.18; 35.99)	Coproscopy
Bedada, H. and F. G. W. Negash (2017b) [43]	Caprine	166	63	37.95 (29.65; 48.59)	Coproscopy
Belete, K. (2017) [44]	Ovine	383	158	41.25 (35.29; 48.21)	Coproscopy
Berhanu, M., et al. (2016) [45]	Ovine	384	189	49.21 (42.68; 56.77)	Coproscopy
Bimirew, F. L. and T. M. Cherinnat (2020) [46]	Ovine	390	177	45.38 (39.16; 52.59)	Coproscopy
Birhanu, A., et al. (2015a) [47]	Ovine	205	53	25.85 (19.76; 33.85)	Coproscopy
Birhanu, A., et al. (2015b) [47]	Caprine	179	19	10.61 (6.78; 16.65)	Coproscopy
Chala, D. and B. Alemu (2019) [48]	Ovine	384	151	39.32(33.53; 46.13)	Coproscopy and Postmortem
Dabasa, G., et al. (2017) [49]	Ovine	384	88	22.91(18.6; 28.25)	Coproscopy
Demissie, T., et al. (2021a) [50]	Ovine	168	28	16.66(11.51; 24.13)	Coproscopy and Postmortem
Demissie, T., et al. (2021b) [50]	Caprine	216	26	12.03(8.19; 17.68)	Coproscopy and Postmortem
Destaw, K., et al. (2017) [51]	Ovine	384	139	36.19(30.66; 42.75)	Coproscopy
Getabalew, M., et al (2019) [52]	Ovine	121	85	70.24(56.8; 86.89)	Coproscopy
Ibrahim, A., et al. (2017) [53]	Ovine	384	164	42.7(36.65; 49.78)	Coproscopy
Jarso D, e. a. (2016) [54]	Ovine	475	229	48.21(42.35; 54.88)	Coproscopy and Postmortem
Kassye, D., et al. (2017a) [55]	Ovine	202	75	37.12(29.61; 46.56)	Postmortem
Kassye, D., et al. (2017b) [55]	Caprine	194	13	6.7(3.9; 11.55)	Postmortem
Kebede, S. and O. Wakgari (2016) [56]	Ovine	384	214	55.72(48.75; 63.72)	Coproscopy
Megersa, B., et al. (2024a) [57]	Ovine	220	54	24.54(18.8; 32.05)	Coproscopy
Megersa, B., et al. (2024b) [57]	Caprine	112	12	10.71(6.08; 18.87)	Coproscopy
Melkamu, S. and M. Asrat (2015) [58]	Ovine	384	137	35.67(30.17; 42.18)	Coproscopy
Mengistu, B. M., et al. (2019a) [59]	Ovine	245	59	24.08(18.66; 31.09)	Coproscopy
Mengistu, B. M., et al. (2019b) [59]	Caprine	139	8	5.75(2.88; 11.51)	Coproscopy
Regasa, A., et al. (2022) [60]	Ovine	327	140	42.81(36.27; 50.53)	Coproscopy
Regea, G. and G. Getachew (2021) [61]	Ovine	384	126	32.81(27.56; 39.07)	Coproscopy
Zekarias, T. and T. Bassa (2019) [62]	Ovine	384	69	17.96(14.19; 22.76)	Coproscopy

### 3.3. The pooled prevalence and prediction interval estimate of Fasciolosis in Ethiopia

**Fig 2:**
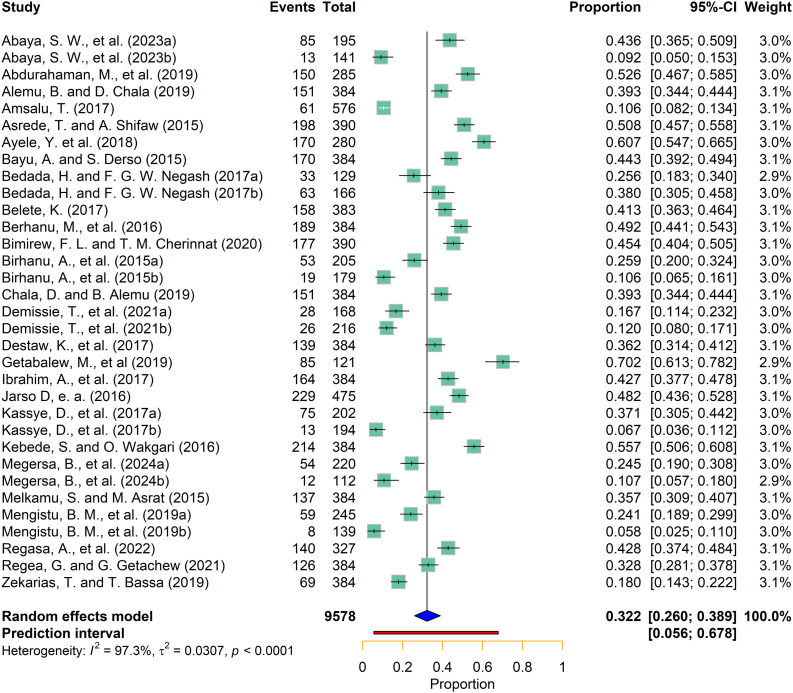
Forest plot for pooled prevalence of Fasciolosis in Ethiopia.

This study identified heterogeneity between the included studies (I^2^ = 97.3% (95% CI: [96.7%; 97.7%], p < 0.001), and τ^2^ = 0.0307 (95% CI: [0.0211; 0.0597], p < 0.001)). As a result, we used a random effects model to estimate the pooled prevalence of Fasciolosis. Based on this model, the pooled effect size of Fasciolosis in small ruminants was 0.3225 (95% CI: [0.2597; 0.3886], with the estimated prediction interval of 5.6–67.8% (Fig 2).

### 3.4. Handling heterogeneity

The meta-analysis indicated that between-study variability was high (Q = 1166.87, df = 32, p < 0.0001), indicating statistically significant differences in effect sizes across the included studies. The variance estimated was τ^2^ = 0.0307 (95% CI: [0.0211; 0.0597], with a *I*^2^ value of (I^2^ = 97.3% (95% CI: [96.7%; 97.7%].

#### 3.4.1. Subgroup analysis.

**Fig 3:**
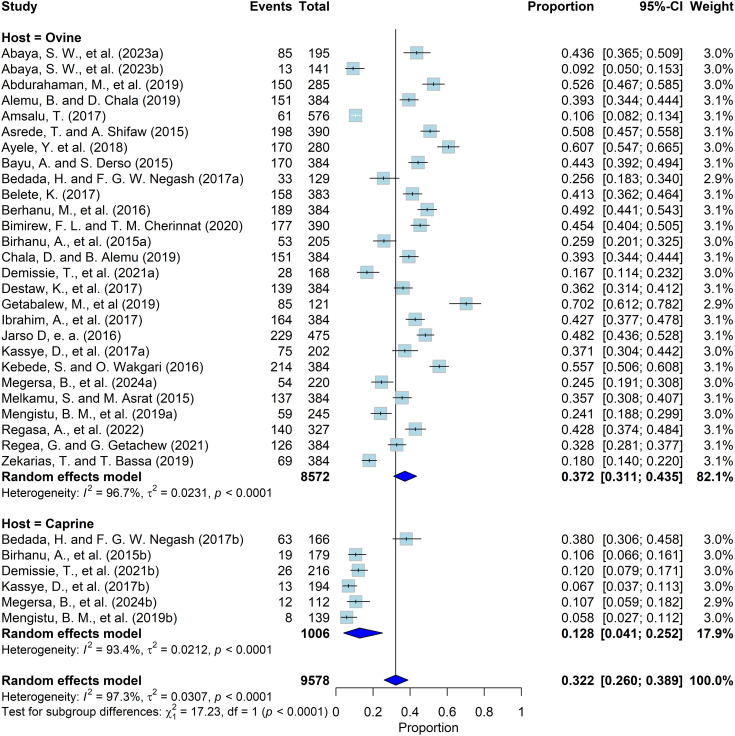
Subgroup analysis based on species of animal.

Subgroup analysis was conducted for the diagnostic methods used (Coproscopy, Postmortem, both Coproscopy and Postmortem, and Immunological test), location of the study, season of data collection, and species of animals studied (Table 2) (Fig 3). Significant variation was observed across region of the study conducted, season, publication year, and animal species. The prevalence of ovine and caprine fasciolosis varied between different regions, ranging from 13.62% to 43.99%. The Amhara region subgroup possessed the highest prevalence (43.99%), while SNNPR region had the lowest prevalence (13.62%). Subgroup analysis based on sampling season showed that the highest infection rates were observed during *Kiremt* (rainy season, June to August) and *Tsedey* (post rainy season, September to November) (52.6%; 95% CI: 46.8–58.4%). The lowest prevalence was observed during *Bega* (dry season, December to February) (25.0%; 95% CI: 14.2–34.9%) (Table 2).

**Table 2:** Subgroup analysis for diagnostic method, region, sampling season, and species of animals studied for fasciolosis.

Variables		Included studies	Proportion (95% CI)	Heterogeneity test (I^2^)	P –value
Regions	Oromia	15	0.3242 [0.2348; 0.4206]	96.5%	0.0006
	SNNPR	2	0.1362 [0.0678; 0.3534]	84.6%	
	Amhara	10	0.4399 [0.3183; 0.5652]	97.9%	
	Afar	2	0.3176 [0.1400; 0.5658]	80.3%	
	Addis Ababa	2	0.1763 [0.0900; 1.2359]	93.4%	
	Tigray	2	0.1376 [0.0598; 0.3120]	96.0%	
Diagnostic Method	Coproscopy	23	0.3488 [0.2778; 0.4234]	96.1%	0.7442
	Postmortem	2	0.1965 [0.1230; 0.3457]	98.3%	
	Coproscopy and Postmortem	6	0.2956 [0.1024; 0.5379]	98.7%	
	Immunological test	2	0.2436 [0.1498; 0.4235]	98.2%	
Sampling season	Bega and Belg	5	0.3944 [0.1656; 0.6509]	96.8%	< 0.0001
	Kiremt and Tsedey	4	0.5263 [0.4681; 0.5841]	94.9%	
	Tsedey and Bega	13	0.3105 [0.2074; 0.4241]	97.8%	
	Bega	11	0.2502 [0.1419; 0.3491]	97.0%	

The subgroup analysis based on species of animal showed that the disease prevalence in ovine was higher (37.18%; 95% CI: [31.06; 43.51]) than in caprine (12.76%; 95% CI: [4.06; 25.19]) (Fig 3).

#### 3.4.2. Sensitivity and influence analysis.

**Fig 4:**
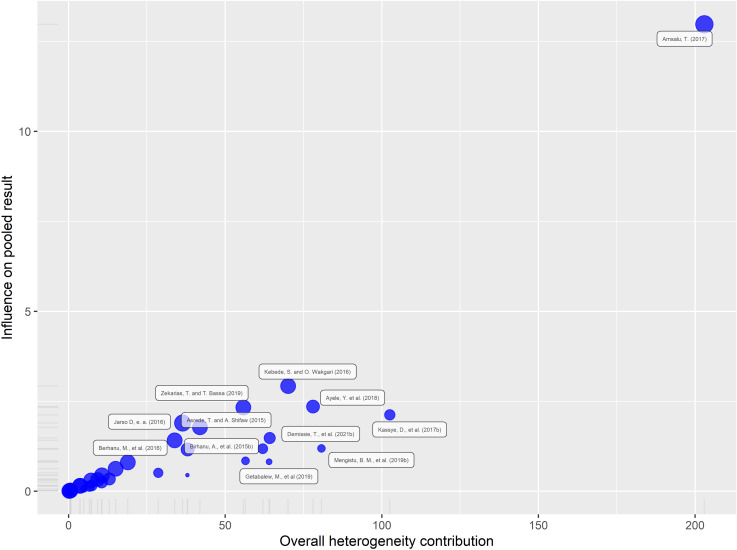
Baujat diagnostic plots of Fasciolosis prevalence among small ruminants in Ethiopia.

Baujat diagnostic analysis was conducted to identify studies that disproportionately contributed to overall heterogeneity. The results indicated that a study by Amsalu, T. (2017) [39] showed a substantial contribution to the observed heterogeneity (Fig 4).

**Fig 5:**
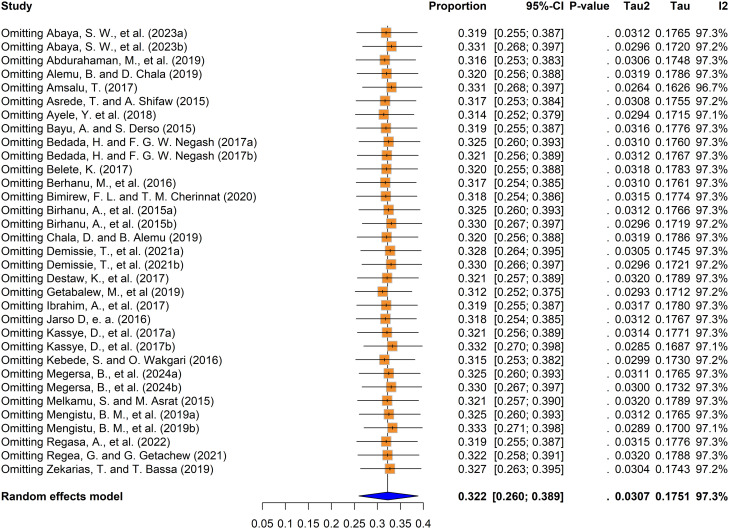
Sensitivity analysis of fasciolosis prevalence among small ruminants in Ethiopia.

A leave-one-out analysis was also performed to assess how each individual study influenced the overall effect size estimate. The meta-analysis produced a pooled effect size of 0.322, which aligned with the confidence intervals of every individual study included. Sensitivity testing further confirmed the stability of these findings, as removing any single study from the analysis did not meaningfully change the overall prevalence estimate (Fig 5). Thus, the meta-analysis results encompassing all included studies in this study were reliable.

#### 3.4.3. Meta-regression models.

Univariable and multivariable meta-regression models were employed to investigate sources of data heterogeneity. In univariable analysis, publication year and sample size were treated as continuous predictors, while study region, species, season, altitude, and diagnostic methods were analyzed as categorical variables within a mixed-effects framework. These predictors were evaluated for linear associations with effect sizes (the dependent variable). Animal species, geographic region, publication year, and sample size demonstrated statistically significant associations with variability across studies. Notably, animal species was found as the strongest explanatory factor, accounting for 25.17% of total heterogeneity (R² = 25.17%). The multivariable model further resolved 39.53% of heterogeneity. The Regression coefficients for multivariable model revealed that studies focusing on Ovine species (β = 0.2913) were associated with elevated effect sizes relative to Caprine studies after adjusting for covariates (Table 3).

**Table 3:** Uni-variable and multivariable meta-regression analysis results for the prevalence of fasciolosis among small ruminants in Ethiopia.

Moderators		^*^R^2^	Coefficient (95% CI)
Univariable regression			
Region	Addis Ababa	12.33%	Reference
	Amhara		0.2897 (0.0151: 0.5643)*
	Oromia		0.1711 (-0.0960: 0.4381)
	SNNPR		-0.0580 (-0.4127: 0.2967)
	Afar		0.1636 (-0.1927: 0.5199)
	Tigray		-0.0520 (-0.4074: 0.3033)
Host	Caprine	25.17%	Reference
	Ovine		0.2876 (0.1383: 0.4369)**
Sample size		6.84%	0.2333 (0.1202: 0.5040)
Publication year		2.27%	-0.0375 (-0.0435: -0.0180)
Altitude	Highlands	0.00%	Reference
	Lowlands		0.0051 (-0.2524: 0.2627)
	Midlands		-0.0508 (-0.1988: 0.0972)
Detection methods	Coproscopy		Reference
	Coproscopy and Postmortem		-0.0561 (0.5387: -0.2405)
	Immunological test		-0.1130 (0.4462: -0.4123)
	Postmortem		-0.1708 (-0.4690: 0.1274)
Multivariable regression			
Full model	**Publication year + Host+ Sample size**	39.53%	
Host	**Ovine**		0.2913 (0.1188: 0.4638)**
Publication year			-0.0165 (-0.0399: 0.0070)
Sample size			-0.0001 (-0.0007: 0.0005)

* R^2^= Coefficient of determination

### 3.5. Publication bias

**Fig 6:**
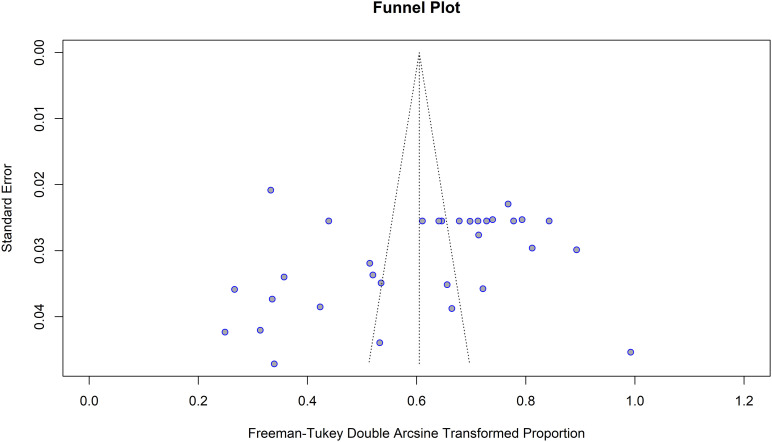
Funnel plot of the pooled prevalence of fasciolosis among small ruminants in Ethiopia.

Publication bias was evaluated through visual and statistical methods [63]. Visual inspection of the funnel plot revealed a symmetrical distribution of study estimates (Fig 6). To complement this subjective assessment, quantitative statistical tests [35] were applied. Egger’s regression analysis (coefficient t = -1.75; p = 0.0906) and Begg’s rank correlation test (z = -2.17; p = 0.0599) both indicated no statistically significant asymmetry in the pooled data. These findings suggest no substantial evidence of publication bias or small-study effects influencing the meta-analytic results.

## 4. Discussion

Fasciolosis, a parasitic infection caused primarily by *Fasciola hepatica* and *Fasciola gigantica*, remains a significant concern in Ethiopia due to its widespread prevalence. In developing nations like Ethiopia, small ruminants, particularly sheep and goats serve as economically vital livestock, supporting livelihoods and food security. This emphasizes the need to synthesize existing data on fasciolosis prevalence and associated risk factors in these animals. To our knowledge, this study represents the first comprehensive SRMA addressing the burden of fasciolosis in Ethiopian small ruminants, alongside evaluation of contributing epidemiological variables. By pooling current evidence, this work aims to support evidence-based interventions and inform targeted control strategies to mitigate the disease’s impact on livestock productivity and rural economies.

The current findings indicate high burden of fasciolosis among small ruminants in Ethiopia. Our finding is larger than earlier studies conducted in China, which reported a prevalence of 26% [64], and a study conducted in Bangladesh, which reported a rate of 20% [65], which reflects epidemiological differences of the disease. The increased prevalence of fasciolosis can be attributed to favorable ecological conditions for the intermediate host [15], traditional management and husbandry practices [66], limited veterinary interventions and drug resistance [67], and differences in diagnostic methodologies that may affect reported prevalence rates [68].

This study demonstrates significant geographic and species-specific variations in fasciolosis prevalence. The result of subgroup analysis by region showed that the highest prevalence of sheep and goats fasciolosis was 43.99% [95% CI: 31.83; 56.52%] in Amhara region, and the lowest prevalence was observed in SNNPR (13.62% [95% CI: 6.78; 35.34%]). The significant variation in fasciolosis prevalence observed across regions and species may be explained by ecological, climatic, and husbandry-related factors that influence the transmission dynamics of the parasite. The Amhara region, characterized by high-altitude wetlands, perennial water bodies, and intensive irrigation systems (e.g., Lake Tana basin), provides optimal habitats for the intermediate hosts of *Fasciola* species [57].

In this study, the prevalence of fasciolosis was observed to be significantly higher in sheep compared to goats, suggesting species-specific susceptibility to the disease. This disparity may stem from differences in grazing behavior and physiological susceptibility. Sheep are known to graze closer to the ground and in wetter, marshy areas where *Fasciola* spp. larvae are more prevalent, increasing their exposure to infective metacercariae [69,70]. In contrast, goats exhibit more selective browsing behavior, often feeding on shrubs and higher vegetation, which may reduce their risk of infection [71]. Additionally, goats may exhibit greater resistance to fasciolosis due to variations in immune response or liver enzyme activity, as suggested by Molina-Hernández et al. (2015) [72]. These findings highlight the need for targeted control measures, particularly in sheep, to mitigate the impact of fasciolosis.

Seasonal variation in fasciolosis prevalence was shown, with significantly higher infection rates during the rainy and post-rainy seasons (*Kiremt* and *Tsedey*), compared to the lowest rates observed in the dry season (*Bega*) (Table 2). This could be attributed to Ethiopia’s unique climatic and ecological drivers. Infection rates peaked during *Kiremt* and *Tsedey,* coinciding with periods of elevated rainfall and humidity. These conditions promote intermediate host proliferation by creating saturated grazing areas and standing water bodies that serve as ideal habitats [73]. Additionally, moisture retention during *Tsedey* sustains vegetation contamination by metacercariae, prolonging livestock exposure [15]. In contrast, the arid *Bega* season reduces transmission risk through habitat desiccation and cooler highland temperatures, which suppress snail activity and metacercarial survival. Shifts in livestock management, such as restricted grazing and stall-feeding during *Bega*, further limit contact with infected pastures [17].

Although this study primarily focused on small ruminants, fasciolosis holds considerable public health importance because of its zoonotic nature. Human fascioliasis, caused by the ingestion of metacercariae-contaminated water or vegetation, has been reported in various regions of Ethiopia, particularly in areas with a close human-livestock-water interface [36]. Livestock infection contributes to environmental contamination and increases the risk of spillover to humans, especially in communities that rely on open water sources shared with livestock and raw vegetation consumption. Furthermore, the economic consequences of fascioliasis, including reduced productivity, liver condemnation, and treatment costs, indirectly affect household income and food security [74,75]. Addressing fascioliasis through integrated One Health approaches linking veterinary, medical, and environmental sectors is therefore critical for reducing both animal and human health risks.

However, this SRMA has some limitations. First, the uneven distribution of studies across various regions of the country, with some regions entirely unrepresented, may affect the interpretation of the pooled prevalence of fascioliasis in Ethiopia and prevent meaningful regional comparisons. In addition, this review prioritized studies published after 2015 to reflect recent epidemiological trends; however, this approach may exclude historical data that are essential for understanding long-term shifts in the burden of fasciolosis.

## 5. Conclusions

This SRMA highlights fasciolosis as a significant threat to Ethiopian small ruminants, with a national pooled prevalence of 32.25%. The elevated burden in Ethiopia reflects the confluence of ecological, climatic, and management factors, including optimal conditions for *Lymnaea* snail proliferation, traditional grazing practices, and limited veterinary interventions. Policymakers and veterinarians should prioritize evidence-based control programs in regions with high disease burden, through seasonal deworming, pasture rotation to disrupt snail ecology, and improved veterinary access in underserved areas. Future research should focus on breed-specific resistance, longitudinal assessments of the climatic impact on disease transmission, and address regional data gaps through improved diagnostic access and targeted epidemiological studies.

## Supporting information

S1 Table PRISMA 2020 checklist. (DOCX)

S2 Table JBI quality appraisal checklist for included studies. (DOCX)

S1 Data Extracted data set (.dta) from selected articles. (DTA)
