# Supplementary material for: Prevalence and risk factors of sheep and goats fasciolosis in Ethiopia: A systematic review and meta-analysis
Source: PLoS Negl Trop Dis. 2025 Aug 18;19(8):e0013074. doi: 10.1371/journal.pntd.0013074 (PMC12373275; doi:10.1371/journal.pntd.0013074)
Supplement: S2 Table — (DOCX) [file pntd.0013074.s002.docx]

**S2 Table. JBI quality appraisal checklist for included studies**

| **JBI Appraisal Checklist for cross-sectional Studies** | | | | | | | | | | | |
| --- | --- | --- | --- | --- | --- | --- | --- | --- | --- | --- | --- |
| Study ID | Q1 | Q2 | Q3 | Q4 | Q5 | Q6 | Q7 | Q8 | Q9 | Total | **Quality** |
| Abaya, S. W., et al. (2023a) [1] | Yes | Yes | Yes | Yes | Yes | Yes | Yes | Yes | NA | 8/9 | High |
| Abaya, S. W., et al. (2023b) [1] | Yes | Yes | Yes | Yes | Yes | Yes | Yes | Yes | NA | 8/9 | High |
| Kiros, A., et al. (2016a) [2] | No | No | No | Yes | No | Yes | Yes | No | NA | 3/9 | Poor |
| Kiros, A., et al. (2016b) [2] | No | No | No | Yes | No | UC | Yes | No | NA | 2/9 | Poor |
| Abdurahaman, M., et al. (2019) [3] | Yes | Yes | No | Yes | No | Yes | Yes | No | NA | 5/9 | Medium |
| Alemu, B. and D. Chala (2019) [4] | Yes | No | Yes | Yes | No | Yes | Yes | No | NA | 5/9 | Medium |
| Amsalu, T. (2017) [5] | Yes | Yes | Yes | Yes | Yes | Yes | Yes | Yes | NA | 7/9 | High |
| Kelemework, S., et al. (2016a) [6] | No | No | Yes | Yes | No | Yes | No | No | NA | 3/9 | Poor |
| Asrede, T. and A. Shifaw (2015) [7] | Yes | No | Yes | Yes | No | Yes | Yes | No | NA | 5/9 | Medium |
| Ayele, Y. et al. (2018) [8] | Yes | Yes | Yes | Yes | Yes | Yes | Yes | No | NA | 7/9 | High |
| Bayu, A. and S. Derso (2015) [9] | No | Yes | Yes | Yes | Yes | Yes | Yes | Yes | NA | 7/9 | High |
| Metages, Y., et al. (2018a) [10] | No | No | Yes | Yes | No | Yes | No | No | NA | 3/9 | Poor |
| Metages, Y., et al. (2018b) [10] | No | No | Yes | Yes | No | Yes | No | No | NA | 3/9 | Poor |
| Bedada, H. and F. G. W. Negash (2017a) [11] | No | Yes | Yes | Yes | Yes | Yes | Yes | Yes | NA | 7/9 | High |
| Kelemework, S., et al. (2016b) [6] | No | No | Yes | Yes | No | UC | No | No | NA | 2/9 | Poor |
| Bedada, H. and F. G. W. Negash (2017b) [11] | No | Yes | Yes | Yes | Yes | Yes | Yes | Yes | NA | 7/9 | High |
| Belete, K. (2017) [12] | No | Yes | Yes | Yes | Yes | Yes | Yes | No | NA | 6/9 | Medium |
| Misgia, F., et al. (2016) [13] | No | No | Yes | Yes | UC | UC | UC | No | NA | 2/9 | Poor |
| Berhanu, M., et al. (2016) [14] | No | No | Yes | Yes | Yes | Yes | Yes | No | NA | 5/9 | Medium |
| Oljira, W., et al. (2022a) [15] | No | No | No | Yes | UC | UC | Yes | UC | NA | 2/9 | Poor |
| Oljira, W., et al. (2022b) [15] | No | No | No | Yes | Yes | UC | UC | UC | NA | 2/9 | Poor |
| Bimirew, F. L. and T. M. Cherinnat (2020) [16] | No | No | Yes | Yes | Yes | Yes | Yes | Yes | NA | 6/9 | Medium |
| Tikuye, S. (2017a) [17] | No | No | No | Yes | No | Yes | Yes | No | NA | 3/9 | Poor |
| Tikuye, S. (2017b) [17] | No | No | No | Yes | No | Yes | Yes | No | NA | 3/9 | Poor |
| Birhanu, A., et al. (2015a) [18] | No | No | No | Yes | Yes | Yes | Yes | No | NA | 4/9 | Medium |
| Birhanu, A., et al. (2015b) [18] | No | No | No | Yes | Yes | Yes | Yes | No | NA | 4/9 | Medium |
| Chala, D. and B. Alemu (2019) [19] | No | No | Yes | Yes | Yes | Yes | Yes | No | NA | 5/9 | Medium |
| Tsegaye, B. and B. Gebeyehu (2015) [20] | No | No | No | Yes | No | Yes | Yes | No | NA | 3/9 | Poor |
| Dabasa, G., et al. (2017) [21] | No | No | Yes | Yes | Yes | Yes | Yes | No | NA | 5/9 | Medium |
| Demissie, T., et al. (2021a) [22] | No | No | No | Yes | Yes | Yes | Yes | No | NA | 4/9 | Medium |
| Demissie, T., et al. (2021b) [22] | No | No | No | Yes | Yes | Yes | Yes | No | NA | 4/9 | Medium |
| Destaw, K., et al. (2017) [23] | Yes | Yes | Yes | Yes | Yes | Yes | Yes | No | NA | 7/9 | High |
| Elemo, K. K. and M. A. Geresu (2017) [24] | No | Yes | No | Yes | Yes | UC | UC | No | NA | 2/9 | poor |
| Getabalew, M., et al (2019) [25] | No | Yes | No | Yes | Yes | Yes | Yes | No | NA | 5/9 | Medium |
| Ibrahim, A., et al. (2017) [26] | Yes | Yes | Yes | Yes | Yes | Yes | Yes | No | NA | 7/9 | High |
| Nuqus, D. T., et al. (2022) [27] | No | No | No | Yes | UC | UC | UC | No | NA | 1/9 | Poor |
| Jarso D, e. a. (2016) [28] | No | No | Yes | Yes | Yes | Yes | Yes | No | NA | 5/9 | Medium |
| Kassye, D., et al. (2017a) [29] | Yes | Yes | Yes | Yes | Yes | Yes | Yes | No | NA | 7/9 | High |
| Kassye, D., et al. (2017b) [29] | Yes | Yes | Yes | Yes | Yes | Yes | Yes | No | NA | 7/9 | High |
| Kebede, S. and O. Wakgari (2016) [30] | No | Yes | Yes | Yes | Yes | Yes | Yes | No | NA | 6/9 | Medium |
| Lakew, Z. and A. Alebie (2015a) | No | No | No | Yes | No | UC | Yes | No | NA | 2/9 | Poor |
| Lakew, Z. and A. Alebie (2015b) [31] | No | No | No | Yes | No | Yes | Yes | No | NA | 3/9 | Poor |
| Megersa, B., et al. (2024a) [32] | Yes | Yes | Yes | Yes | Yes | Yes | Yes | Yes | NA | 8/9 | High |
| Megersa, B., et al. (2024b) [32] | Yes | Yes | Yes | Yes | Yes | Yes | Yes | Yes | NA | 8/9 | High |
| Megerssa, Y., et al. (2017) [33] | No | No | No | Yes | Yes | Yes | No | No | NA | 3/9 | Poor |
| Melkamu, S. and M. Asrat (2015) [34] | No | No | Yes | Yes | Yes | Yes | Yes | No | NA | 5/9 | Medium |
| Mengistu, B. M., et al. (2019a) [35] | No | No | No | Yes | Yes | Yes | Yes | No | NA | 4/9 | Medium |
| Mengistu, B. M., et al. (2019b) [35] | No | No | No | Yes | Yes | Yes | Yes | No | NA | 4/9 | Medium |
| Mensur, S., et al. (2016a) [36] | No | No | Yes | Yes | No | Yes | No | No | NA | 3/9 | Poor |
| Mensur, S., et al. (2016b) [36] | No | No | Yes | Yes | No | UC | No | No | NA | 2/9 | Poor |
| Regasa, A., et al. (2022) [37] | No | No | Yes | Yes | Yes | Yes | Yes | Yes | NA | 6/9 | Medium |
| Regea, G. and G. Getachew (2021) [38] | No | No | No | Yes | Yes | Yes | Yes | No | NA | 4/9 | Medium |
| Sebro, E., et al. (2022a) [39] | No | No | No | Yes | No | Yes | Yes | No | NA | 3/9 | Poor |
| Sebro, E., et al. (2022b) [39] | No | No | No | Yes | No | Yes | Yes | No | NA | 3/9 | Poor |
| Zekarias, T. and T. Bassa (2019) [40] | No | No | Yes | Yes | Yes | Yes | Yes | No | NA | 5/9 | Medium |

**Explanation of Prevalence Critical Appraisal [41]**

Answers: Yes, No, Unclear (UC) or Not/Applicable (NA)

1. **Was the sample frame appropriate to address the target population?**

This question relies upon knowledge of the ***broader characteristics of the population of interest and the geographical area.*** If the study is of women with breast cancer, knowledge of at least the characteristics, demographics and medical history is needed. The term “target population” should not be taken to infer every individual from everywhere or with similar disease or exposure characteristics. Instead, give consideration to specific population characteristics in the study, including age range, gender, morbidities, medications, and other potentially influential factors. For example, a sample frame may not be appropriate to address the target population if a certain group has been used (such as those working for one organization, or one profession) and the results then inferred to the target population (i.e. working adults). A sample frame may be appropriate when it includes almost all the members of the target population (i.e. a census, or a complete list of participants or complete registry data).

1. **Were study participants recruited in an appropriate way?**

Studies may report ***random sampling from a population*,** and the methods section should report how sampling was performed. Random probabilistic sampling from a defined subset of the population (sample frame) should be employed in most cases, however, random probabilistic sampling is not needed when everyone in the sampling frame will be included/ analysed. For example, reporting on all the data from a good census is appropriate as a good census will identify everybody. When using cluster sampling, such as a random sample of villages within a region, the methods need to be clearly stated as the precision of the final prevalence estimate incorporates the clustering effect. ***Convenience samples,*** such as a street survey or interviewing lots of people at public gatherings are not considered to provide a representative sample of the base population.

1. **Was the sample size adequate?**

The larger the sample, the narrower will be the confidence interval around the prevalence estimate, making the results more precise. An adequate sample size is important to ensure good precision of the final estimate. Ideally, we are looking for evidence that the authors conducted a sample size calculation to determine an adequate sample size. This will estimate how many subjects are needed to produce a reliable estimate of the measure(s) of interest. For conditions with a low prevalence, a larger sample size is needed. Also consider sample sizes for subgroup (or characteristics) analyses, and whether these are appropriate. Sometimes, the study will be large enough (as in large national surveys) whereby a sample size calculation is not required. In these cases, sample size can be considered adequate.

When there is no sample size calculation and it is not a large national survey, the reviewers may consider conducting their own sample size analysis using the following formula: (Naing et al. 2006, Daniel 1999)

$$n= \frac{Z^{2}P(1-P)}{d^{2}}$$

Where: n= sample size

Z = Z statistic for a level of confidence

P = Expected prevalence or proportion (in proportion of one; if 20%, P = 0.2) d = precision (in proportion of one; if 5%, d=0.05)

Ref:

Naing L, Winn T, Rusli BN. Practical issues in calculating the sample size for prevalence studies Archives of Orofacial Sciences. 2006; 1:9-14.

Daniel WW. Biostatistics: A Foundation for Analysis in the Health Sciences. Edition. 7th ed. New York: John Wiley & Sons. 1999.

**4. Were the study subjects and setting described in detail?**

Certain diseases or conditions vary in prevalence across different geographic regions and populations (e. g. Women vs. Men, sociodemographic variables between countries). The study sample should be described in sufficient detail so that other researchers can determine if it is comparable to the population of interest to them.

**5. Was data analysis conducted with sufficient coverage of the identified sample?**

***Coverage bias*** can occur when ***not all subgroups of the identified sample respond at the same rate***. For instance, you may have a very high response rate overall for your study, but the response rate for a certain subgroup (i.e. older adults) may be quite low.

**6. Were valid methods used for the identification of the condition?**

Here we are looking for ***measurement or classification bias.*** Many health problems are not easily diagnosed or defined and some measures may not be capable of including or excluding appropriate levels or stages of the health problem. If the outcomes were assessed based on existing definitions or ***diagnostic criteria***, then the answer to this question is likely to be yes. If the outcomes were assessed using observer reported, or self-reported scales, the risk of over- or under-reporting is increased, and objectivity is compromised. Importantly, determine if the measurement tools used were validated instruments as this has a significant impact on outcome assessment validity.

**7. Was the condition measured in a standard, reliable way for all participants?**

Considerable judgment is required to determine the presence of some health outcomes. Having established the validity of the outcome measurement instrument (see item 6 of this scale), it is important to establish how the measurement was conducted. Were those involved in ***collecting data trained or educated in the use of the instrument/s***? If there was more than one data collector, were they similar in terms of level of education, clinical or research experience, or level of responsibility in the piece of research being appraised? When there was more than one observer or collector, was there comparison of results from across the observers? Was the condition measured in the same way for all participants?

**8. Was there appropriate statistical analysis?**

Importantly, the numerator and denominator should be clearly reported, and ***percentages should be given with confidence intervals.*** The methods section should be detailed enough for reviewers to identify the analytical technique used and how specific variables were measured. Additionally, it is also important to assess the appropriateness of the analytical strategy in terms of the assumptions associated with the approach as differing methods of analysis are based on differing assumptions about the data and how it will respond.

**9. Was the response rate adequate, and if not, was the low response rate managed appropriately?**

A large number of dropouts, refusals or “not founds” amongst selected subjects may diminish a study’s validity, as can a low response rates for survey studies. The authors should clearly discuss the response rate and any reasons for non-response and compare persons in the study to those not in the study, particularly with regards to their socio-demographic characteristics. If reasons for non-response appear to be unrelated to the outcome measured and the characteristics of non-responders are comparable to those who do respond in the study (addressed in question 5, coverage bias), the researchers may be able to justify a more modest response rate.

# Reference

1. Abaya, S.W., S.T. Mereta, F.D. Tulu, Z. Mekonnen, M. Ayana, M. Girma, H.R. Vineer, S.M. Mor, C. Caminade, and J. Graham-Brown, *Prevalence of human and animal fasciolosis in Butajira and Gilgel Gibe health demographic surveillance system sites in Ethiopia.* Tropical Medicine and Infectious Disease, 2023. **8**(4): p. 208.

2. Kiros, A., B. Tadesse, A. Aylate, and B. Tadesse, *Prevalence of Fasciolosis in small ruminants and associated risk factor in and around Kombolcha.* J. Adv. Parasitol, 2016. **3**(2): p. 61-65.

3. Abdurahaman, M., T. Dinagde, T. Kedir, T. Ahimad, T. Said, T. Mamo, and H. Tesfaye, *A Study on Prevalence of Ovine Fasciolosis in Busa Town, Dawo Woreda, South West Shoa Zone, Oromia Region.* International Journal of Research, 2019. **7**(3): p. 1-6.

4. Alemu, B. and D. Chala, *Prevalence of ovine fasciolosis and its economic impacts in and around Ambo, Ethiopia.* International Journal, 2019. **5**(1): p. 033-038.

5. Amsalu, T., *Prevalence of ovine fasciolosis and loss due to liver condemnation at Bahir Dar Town, Ethiopia.* Adv. Biol. Res, 2017. **11**(5): p. 286-294.

6. Kelemework, S., A. Tilahun, E. Benalfew, and A. Getachew, *A study on prevalence of gastrointestinal helminthiasis of sheep and goats in and around Dire Dawa, Eastern Ethiopia.* Journal of Parasitology and Vector Biology, 2016. **8**(10): p. 107-113.

7. Asrede, T. and A. Shifaw, *Coprological Study on the prevalence of Ovine Fasciolosis in Debre Birhan Agricultural Research Center, Ethiopia.* Eur J Biol Sci, 2015. **7**: p. 103-7.

8. Ayele Y., w.F., and Yeshiwas T, *The Prevalence of Bovine and Ovine Fasciolosis and the Associated Economic Loss Due to Liver Condemnation in and around Debire Birhan, Ethiopia. .* SOJ Immunol 2018. **6 (3): 1-11**.

9. Bayou, K. and T. Geda, *Prevalence of bovine fasciolosis and its associated risk factors in Haranfama municipal abattoir, Girja District, South-Eastern Ethiopia.* Int J Anim Sci, 2018. **2**(2): p. 1018s1.

10. Metages, Y., M. Mathewos, A. Ademasu, and E. Mikias, *Abatoir Survey on Prevalence and Economic Significance of Fasciolosis in Small Ruminants Slaughtered in Addis Ababa Municipal Abatoir, Ethiopia.* Acta Parasitol, 2018. **9**: p. 112-120.

11. Bedada, H., F. Gizaw, W. Negash, A. Hadush, A. Wassie, and A. Gebregergious, *Epidemiology of Small Ruminant Fasciolosis in Arid Areas of Lower Awash River Basin, Afar Region, Ethiopia.* Animal and Veterinary Sciences, 2017. **5**(6): p. 102-107.

12. Belete, K., *A cross sectional study on the coprological prevalence of ovine fasciolosis in Amhara Sayint District, Ethiopia.* Journal of Veterinary Medicine Research, 2017. **4**(6): p. 1092.

13. Misgia, F., B. Basaznew, and T. Shimels, *Coporological Prevalence of Ovine Fasciolosis in and Around Debre-Tabor Town, Ethiopia.* European Journal of Biological Sciences, 2016. **8**(2): p. 60-63.

14. Berhanu, M., G. Ayalew, and A. Tilahun, *Prevalence and Associated Risk Factors for Ovine Fasciolosis in Selected Areas of North Gondar, Ethiopia.* Advances in Biological Research, 2016. **10**(3): p. 162-166.

15. Oljira, W., B. Mideksa, G. Mekonnen, G. Kebebew, and E. Jorga, *Fasciolosis in sheep and goats slaughtered at abattoirs in Central Ethiopia and associated financial losses.* Food and Waterborne Parasitology, 2022. **28**: p. e00173.

16. Bimirew, F.L. and T.M. Cherinnat, *Coprological Examination of Ovine Fasciolosis in Horro District Community Based Sheep Breeding Program, Horro Guduru Wollega Zone, Western Ethiopia.* Journal of Veterinary Healthcare, 2020. **2**(2): p. 31-38.

17. Tikuye, S., *Study on prevalence of ruminant fasciolosis and its associated risk factors in Kombolcha, North East Ethiopia.* J Vet Sci Technol, 2017. **8**(4): p. 1000461.

18. Birhanu, A., R. Tesfaye, and S. Derso, *Prevalence and associated risk factors of Fasciola infection in small ruminants slaughtered at Addis Ababa abattoir Enterprise, Ethiopia with reference to diagnostic value of its coprological examination.* African Journal of Basic & Applied Sciences, 2015. **7**(4): p. 181-186.

19. Chala, D. and B. Alemu, *Prevalence of Ovine Fasciolosis and Its Economic Loss In and Around Ambo, Ethiopia.* 2019.

20. Tsegaye, B. and B. Gebeyehu, *Fasciolosis: Abattoir prevalence and severity of liver lesions in sheep slaughtered at Debre-Birhan Municipal Abattoir, North East Ethiopia.* Int. J. Agric. Sci. Res, 2015. **4**: p. 78-81.

21. Dabasa, G., T. Shanko, W. Zewdei, K. Jilo, G. Gurmesa, and N. Abdela, *Prevalence of small ruminant gastrointestinal parasites infections and associated risk factors in selected districts of Bale zone, south eastern Ethiopia.* Journal of Parasitology and Vector Biology, 2017. **9**(6): p. 81-88.

22. Demissie, T., B. Wakjira, J. Shiferaw, A. Feyisa, and Y.H. Tolossa, *Pre-slaughter coproscopic and abattoir prevalence of GIT parasites of sheep and goats at Bishoftu ELFORA export abattoir, Oromia, Ethiopia.*

23. Destaw, K., W. Mitku, M. Hamid, B. Alemu, and T. Tintagu, *Prevalence of ovine fasciolosis in selected Kebeles of Wogera District, North Gondar zone, Ethiopia.* Int. J. Adv. Res. Biol. Sci, 2017. **4**(8): p. 78-84.

24. Elemo, K.K. and M.A. Geresu, *Prevalence and Risk Factors of Gastro Intestinal Parasitesof Small Ruminants in Sinana and Dinsho Districts of Bale Zone, South Eastern Ethiopia.* European Journal of Biological Sciences, 2017. **9**(1): p. 01-08.

25. Getabalew, M., T. Alemneh, D. Akeberegn, A. Getie, B. Fekadu, H. Hadgu, and N. Zeselase, *Prevalence of Ovine Fasciolosis in Debre Berhan Agricultural Research Center, North Shewa Zone, Ethiopia.*

26. Ibrahim, A., D. Nolkes, E. Gezahegn, and M. Taye, *Prevalence of ovine fasciolosis in Jimma and selected rural kebeles near Jimma, Southwest Ethiopia.* J Vet Sci Technol, 2017. **8**(424): p. 2.

27. Nuqus, D.T., D.A. Wako, and J.D. Kitessa, *Prevalence of ovine fasciolosis and its associated risk factors: The case of in and around Bedele Town, Bunno Bedele Zone, Oromia Region, Ethiopia.* International Journal of Veterinary Science and Research, 2022. **8**(2): p. 74-79.

28. Jarso D, e.a., *Study on Prevalence of Ovine Fasciolosis in and Around Debre Berhan Sheep Breeding and Forage Multiplication Center.* Journal of Veterinary Science & Research, 2016.

29. Kassye, D., M. Gebeyehu, and D. Mekonnen, *Prevalence and associated risk factors of small ruminant Fasciolosis in Haramaya district, Eastern Ethiopia.* Acta Parasitol, 2017. **8**: p. 144-149.

30. Kebede, S. and O. Wakgari, *The prevalence study of ovine fasciolosis in Jima Rare District, Horo Guduru Wollega Zone, Oromia Regional State, Western Ethiopia.* 2016.

31. Lakew, Z. and A. Alebie, *A Study on the Prevalence, Risk Factors and Financial Lose of Cattle, Sheep and Goats Fasciolosis in Debrezeit Town, Ethiopia.* American-Eurasian Journal of Scientific Research, 2015. **10**(3): p. 126-133.

32. Megersa, B., B. Hussien, J. Shemsu, R. Kassahun, O. Merera, N. Moje, B.M. Edao, H. Ashenafi, and D. Ayana, *Trematode Infestations in Ruminants and Their Snail Hosts across varied Agro-Ecological zones in Ethiopia: Implication for Public Health Risk.* 2024.

33. Megerssa, Y., T. Jima, and Y. Diriba, *W/Mariyam FT (2017) Burden of Ovine Fasiolosis in Sherka Woreda Arsi, Ethiopia.* J Vet Sci Technol. **8**(426): p. 2.

34. Melkamu, S. and M. Asrat, *Study on the prevalence of Ovine fasciolosis in Ambasel Woreda, South Wollo zone, Amhara regional state, Ethiopia.* Journal of Animal Research, 2015. **5**(3): p. 437-441.

35. Mengistu, B.M., A.A. Azbite, and H.K. Bitsue, *Prevalence of Small Ruminants Fasciolosis in Mekelle, Tigrai Regional State, Northern Ethiopia.* Journal of Agriculture and Ecology Research International, 2019. **20**(1): p. 1-8.

36. Mensur, S., I. Ansuar, A. Tesfaye, K. Abdulkaf, and Y. Ahmed, *Small ruminant fasciolosis and its economic impact in an export abattoir of Ethiopia.* Livestock Research for Rural Development, 2016. **28**: p. 9.

37. Regasa, A., B. Mesfin, and M. Adem, *Study on prevalence and risk factors of Ovine Fasciolosis on Shirka District, Arsi Zone, Eastern Ethiopia.* J Vet Med Heal, 2022. **6**: p. 2.

38. Regea, G. and G. Getachew, *Study on prevalence and associated risk factors of ovine fasciolosis in and around Nekemte Town, Oromia, Ethiopia.* Journal of Veterinary Medicine and Animal Sciences, 2021. **4**(1).

39. Sebro, E., M. Kebamo, and A. Abebe, *Prevalence of gastrointestinal parasites of sheep and goats in an-Lemo, Hadiya zone southern Ethiopia.* Indian Journal of Science and Technology, 2022. **15**(22): p. 1084-1090.

40. Zekarias, T. and T. Bassa, *Prevalence of Ovine Fasciolosis in Damot Sore Woreda, Wolayta Zone, Ethiopia.* 2019.

41. Munn, Z., S. Moola, K. Lisy, D. Riitano, and C. Tufanaru, *Methodological guidance for systematic reviews of observational epidemiological studies reporting prevalence and cumulative incidence data.* JBI Evidence Implementation, 2015. **13**(3): p. 147-153.
